# Appendiceal Adenocarcinoma Masquerading as a Chronic Intra-abdominal Abscess

**DOI:** 10.7759/cureus.103054

**Published:** 2026-02-05

**Authors:** Sabeen Wazir, Lydia Ta, Jude Aguillard, Savannah Newell, Stephen C Wheelis

**Affiliations:** 1 Medicine, Edward Via College of Osteopathic Medicine, Monroe, USA; 2 Anatomical Sciences, Edward Via College of Osteopathic Medicine, Monroe, USA; 3 General Surgery, CHRISTUS St. Frances Cabrini Hospital/Edward Via College of Osteopathic Medicine, Alexandria, USA

**Keywords:** appendiceal adenocarcinoma, appendiceal phlegmon, autosomal dominant polycystic kidney, malignancy, supportive and palliative care

## Abstract

Appendiceal adenocarcinoma is a rare gastrointestinal malignancy that often presents with nonspecific symptoms and is frequently misdiagnosed as acute or chronic appendicitis. This condition is often characterized by chronic lower right quadrant abdominal pain, bloating, changes in bowel habits, and weight loss. Diagnostic delay is common, particularly in patients managed conservatively or those with comorbid conditions that obscure abdominal symptomatology. We present a case of appendiceal adenocarcinoma discovered following recurrent appendicitis complicated by chronic intra-abdominal abscess in an 81-year-old man with autosomal dominant polycystic kidney disease (PCKD). Given the patient’s age and comorbidities, palliative care was pursued. This case highlights the diagnostic challenges of appendiceal malignancy, particularly in patients with recurrent appendicitis and PCKD, failed conservative management, and comorbid conditions that may obscure the diagnostic process.

## Introduction

Located in the right lower quadrant of the abdomen, the appendix is a small tube-shaped pouch that is attached to the ascending colon near the ileocecal junction. The role of the appendix is still debated, with historical theories stating it is a vestigial organ and more recent data highlighting its role in aiding gut recolonization after the eradication of the intestinal microbiome. Furthermore, research suggests that the removal of the appendix can increase the chances of additional gastrointestinal diseases [1].

Malignancy of the appendix is rare, accounting for approximately 0.08% of all cancers [2]. Cancers of the appendix can be primarily divided into neuroendocrine tumors and carcinomas. The most common neuroendocrine tumors of the appendix are carcinoid tumors, which typically arise from enterochromaffin cells and are often located at the tip of the appendix [2]. These tumors are usually slow-growing and may be found incidentally during appendectomy, though larger lesions can occasionally cause obstruction or metastasis. In contrast to these indolent neuroendocrine tumors, appendiceal carcinomas are more aggressive and encompass four main histologic types: colonic adenocarcinoma, mucinous adenocarcinoma, goblet cell carcinoids, and signet-ring adenocarcinoma [3]. Patients who present with appendiceal adenocarcinoma (ApAC) typically present with abdominal pain, abdominal bloating/distention, and pelvic pain. Given that an estimated 30% of cases commonly present as acute appendicitis, diagnosing appendiceal cancer can be challenging [4]. This diagnostic delay can cause many challenges in patient care, including the metastasis of the cancer and appendiceal perforation [5].

Certain genetic conditions may predispose one to gastrointestinal malignancies. Polycystic kidney disease (PCKD) is an autosomal dominant disorder characterized by progressive cyst formation in the kidneys. While the association between appendiceal malignancy and PCKD is unknown, this condition may increase the risk of gastrointestinal malignancy [6]. The presence of PCKD may complicate the evaluation of abdominal pain due to overlapping symptoms (right lower quadrant pain, fever, leukocytosis, nausea/vomiting, and abdominal tenderness). As a result, the underlying malignancy may be overlooked or misattributed to the renal disease process.

Due to its rarity, our current knowledge is limited by the lack of literature available to study the appendix. We intend to elaborate on the case of an 81-year-old male patient with PCKD and chronic perforated appendicitis resulting in an intra-abdominal abscess and an ultimate diagnosis of appendiceal carcinoma. This case carries broader clinical relevance by illustrating a diagnostic pitfall that may be encountered in older patients presenting with complicated appendicitis.

## Case presentation

An 81-year-old male presented to the emergency department (ED) with a primary complaint of right lower quadrant abdominal pain. Additional symptoms include low-grade fever (37.6 ℃) and loss of appetite. Past medical history includes end-stage renal disease (ESRD), PCKD, right lower quadrant abdominal abscess, hypertension, type II diabetes mellitus (TIIDM), and normocytic anemia. The patient reported that one year ago, they were diagnosed with an intra-abdominal abscess suspected from a perforated appendix that was managed with a percutaneous drain (PERC) that the patient has since retained.

**Figure 1:**
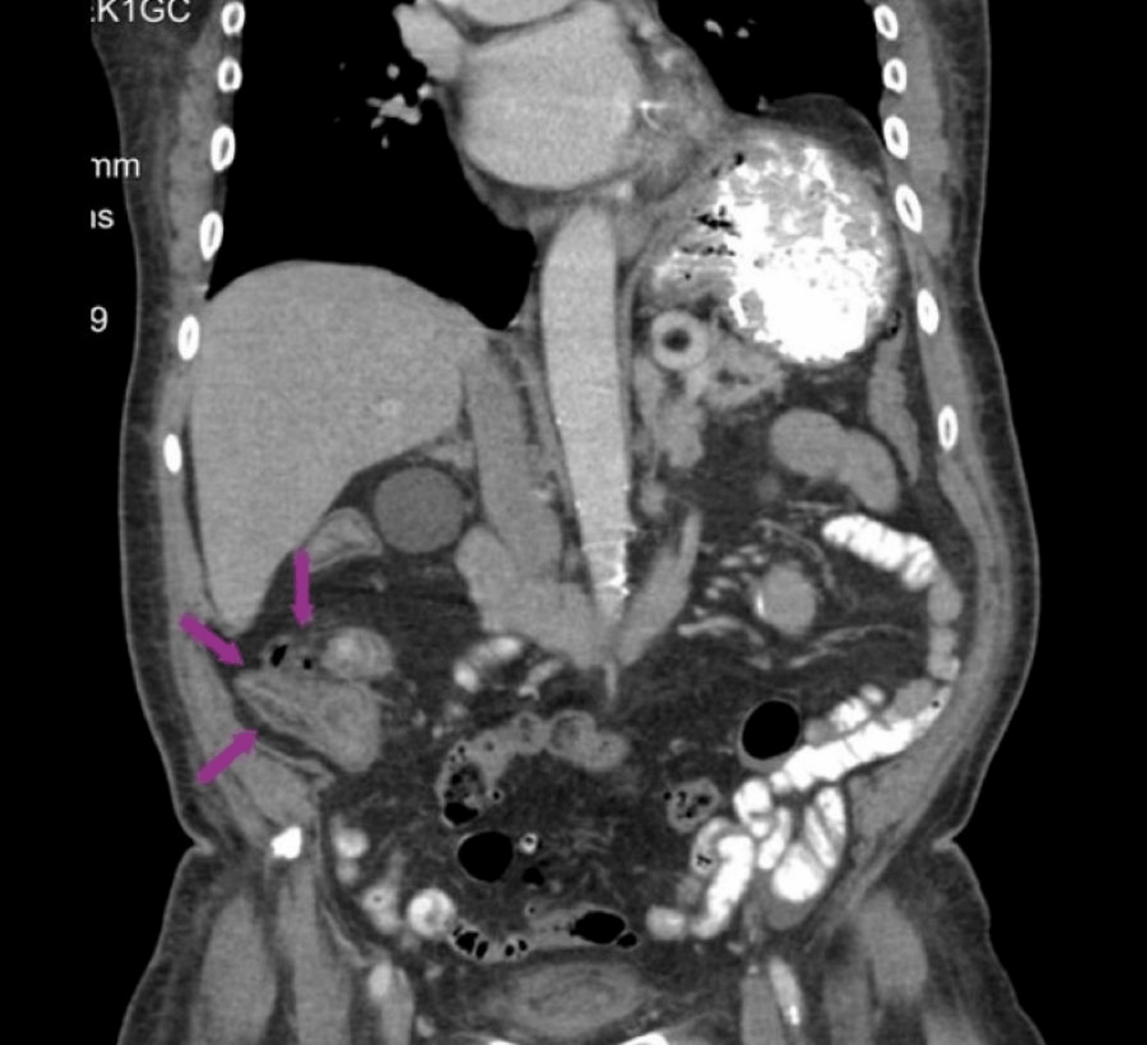
Contrast-enhanced CT of abdomen and pelvis (coronal view) two months prior to current presentation, displaying abscess (purple arrows) of the appendix

Two months before the current presentation to the ED (approximately 10 months following the PERC placement), the patient accidentally pulled out his PERC. At that time, the patient presented to the ED and received a computed tomography (CT) scan of the abdomen and pelvis (Figure 1), which revealed an abscess; however, the PERC was not replaced due to asymptomatic presentation. The patient denied vomiting, diarrhea, and all other symptoms at this time. Physical examination revealed abdominal tenderness in the right lower quadrant with an absence of guarding and rebounding. All other examination findings were normal.

**Figure 2:**
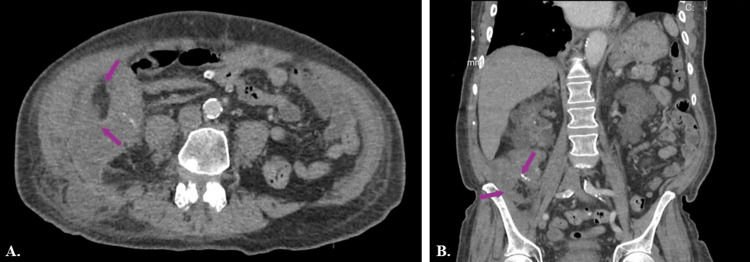
CT of abdomen and pelvis (A) transverse and (B) coronal views Purple arrows indicate right Inflammatory process noted in the right lower abdominal quadrant potentially remnant of the appendix

At the current presentation to the ER, while the patient’s vitals indicated a low-grade fever, the complete blood count (CBC) revealed an elevated white blood cell (WBC) count consistent with infection (Table 1). CT of the abdomen and pelvis without contrast scan indicated the presence of an inflammatory process in the left retroperitoneum near the inferior border of the liver, extending along the right retroperitoneum to the inguinal region. Furthermore, the CT also indicated an inflammatory process in the right colon, which extended through the abdominal wall (Figure 2). Based on the WBC and the CT imaging, the general surgery team consulted the patient and determined the need for drainage of the intra-abdominal abscess via interventional radiology (IR).

**Table 1:** Patient laboratory values: (complete blood count) at emergency department showing high WBC count ↑ = increased value, ↓ = decreased value, and ↑↑ = abnormally high increased value WBC: white blood cell, RBC: red blood cell

Tests	Patient Values	Reference Ranges
WBC	34.5 10*3/uL ↑↑	4.0 - 10.0 10*3/uL
Red Cell Count	3.94 10*6/uL ↓	4.40 - 6.10 10*6/uL
Hemoglobin	10.6 g/dL ↓	13.2 - 16.6 g/dL
Hematocrit	31.3 % ↓	40.1 - 51.0 %
Mean Corpuscular Volume	79.4 fL ↓	80.0 - 100.0 fL
Mean Corpuscular Hemoglobin	26.9 pg	25.6 - 32.2 pg
Mean Corpuscular Hemoglobin Concentration	33.9 g/dL	32.0 - 36.0 g/dL
Red Cell Distribution Width	15.9% ↑	12.0 - 15.0%
Platelet Count	419 10*3/uL ↑	150 - 400 10*3/uL
Mean Platelet Volume	10.3 fL	7.5 - 11.2 fL
Nucleated RBC %	0.0%	0.0 - 1.0%

Four days after the presentation, a contrast-enhanced CT was done to better understand the depth of the abscess. IR and general surgery were unable to drain the abscess due to a mass-like feeling blocking the procedure. The inability to drain the abscess, the high WBC count, and the recurrent appendicitis were concerning for peritonitis.

Upon physical exam the next day, the patient appeared frail and demonstrated immense pain with slight movement. An exploratory laparotomy was conducted to determine the source of infection, which revealed that the patient had thickened inflammation throughout the right lower quadrant, with the cecum firmly adhered to the abdominal wall. Once the adhesions were removed, it was apparent that there was a cecal fistula in the area where the appendix once resided. Unfortunately, the appendix could not be identified as it had likely been obliterated by chronic inflammatory processes. Furthermore, the patient had a large volume of pus in the area and the flank abdominal wall.

**Figure 3:**
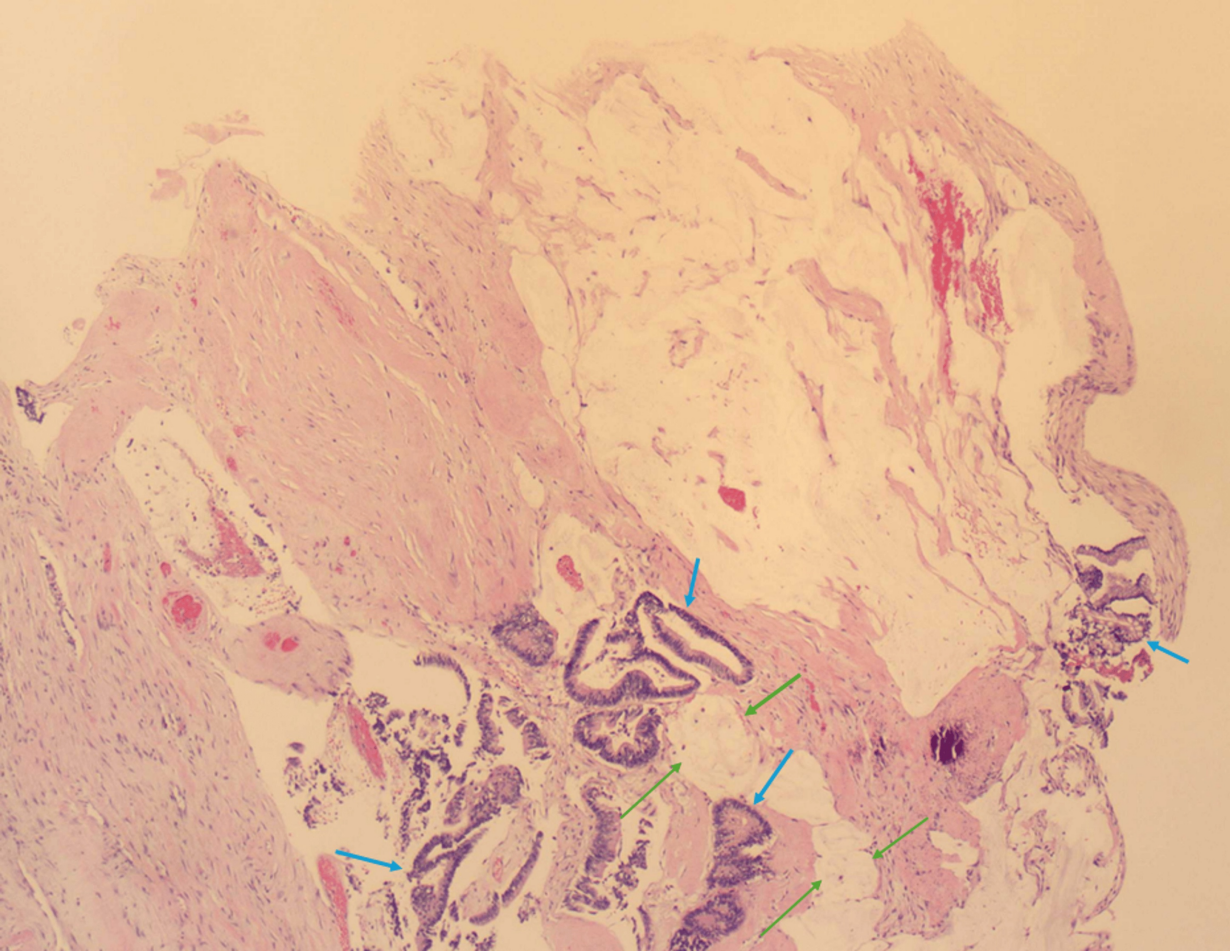
Colonic adenocarcinoma with mucinous features Low power view of cecal-appendiceal junction.  Extracellular mucin pools (green arrows) dissect through the colonic wall with scattered neoplastic glands (blue arrows). These findings are characteristic colonic appendiceal adenocarcinoma with mucinous features.

The terminal ileum and cecum were resected and sent to pathology for analysis. An ileocecal anastomosis reconnected the intestines. Due to the obliteration of the appendix, histology from both the ileum and cecum revealed evidence of a colonic adenocarcinoma, highly suspicious of primary ApAC (Figure 3). The patient was ultimately diagnosed with appendiceal colonic carcinoma. He healed well postoperatively and was given cefepime 1 g intravenously to prevent infection. Referral to oncology was made prior to discharge. Due to the patient’s age and wishes, he was not considered a good candidate for further treatment. Therefore, they opted for palliative care at this time.

## Discussion

There are many challenges to diagnosing appendiceal cancer, including low clinical suspicion, non-specific clinical presentation, and its ability to remain asymptomatic. Appendiceal malignancies comprise less than 1% of colonic cancers [7]. One challenge is this malignancy’s ability to mimic more common colonic conditions, such as acute and chronic appendicitis. It is estimated that 32% and 23% of appendiceal mucinous neoplasm patients were diagnosed with acute appendicitis preoperatively and incidentally, respectively [8] This complex interplay sets appendiceal cancer apart from other colonic malignancies, which typically present with symptoms such as rectal bleeding, anemia, or changes in bowel habits, rather than mimicking a surgical emergency. In this patient, the prolonged course of presumed chronic appendicitis with recurrent abscess formation exemplifies how appendiceal carcinoma can mimic benign inflammatory processes, contributing to delayed recognition of the underlying malignancy.

While poorly understood, research suggests that acute appendicitis treated conservatively may be associated with an increased risk of underlying appendiceal neoplasms, including ApAC. This association may in part reflect the overlapping clinical presentation between appendiceal malignancies and acute appendicitis, which can delay diagnosis when surgical pathology is not obtained [8,9]. Surgical resection of the appendix allows for definitive histopathologic evaluation, excluding occult malignancy. Guidance from the American College of Surgeons supports conservative management with antibiotics in patients with uncomplicated acute appendicitis, emphasizing the importance of ongoing clinical follow-up due to the risk of recurrence and missed pathology [10]. Although conservatively managed appendicitis has been associated with appendiceal cancer, identifying additional risk and prognostic factors for ApAC remains challenging, with existing clinical and imaging predictors demonstrating limited sensitivity and specificity [11]. In this case, initial conservative management of presumed perforated appendicitis without definitive surgical pathology likely contributed to delayed diagnosis, with malignancy only identified after failure of percutaneous drainage and progression of disease.

The relationship between PCKD and appendiceal malignancies is poorly understood, with only a handful of cases reported in the literature and considered an incidental coincidence [12]. Several studies have shown higher rates of colorectal and hepatobiliary cancers in patients with autosomal dominant PCKD (ADPKD), thought to be related to chronic inflammation, altered cellular signaling, and repeated abdominal imaging uncovering incidental lesions [1,13]. Despite their anatomical proximity, appendiceal malignancies are not considered colorectal malignancies, so the research on colorectal malignancies and PCKD technically does not apply to this cancer. However, the absence of research on appendiceal cancer does not rule out the potential for a link between appendiceal cancer and PCKD. Furthermore, a study of two relatives with primary appendiceal cancer revealed that both tested positive for the mutation in the *PKD1* gene and had ADPCKD as well [12].

PCKD is characterized by the progressive development of renal cysts and systemic complications, including hypertension, aneurysms, and chronic kidney disease. From a diagnostic perspective, this may pose challenges due to concurrent symptom presentation underlying PCKD, appendicitis, and appendiceal malignancies. Abdominal pain and bloating are common in these conditions, which may delay recognition of the underlying malignancy [14]. The overlap in symptoms between these two conditions may be a reason why this patient progressed from acute appendicitis to an intra-abdominal abscess. Overlapping abdominal symptoms and complex comorbidities may have further obscured early recognition of appendiceal malignancy, potentially facilitating progression from acute appendicitis to chronic abscess formation as seen in this patient.

This patient’s obliterated appendix makes establishing the primary cancer slightly challenging. However, the clinical presentation, patient history, and the pattern of spread in this patient are highly suggestive of appendiceal origin. Furthermore, appendiceal cancers have been recognized as completely separate entities from colorectal cancers due to their genotype [15]. Appendiceal cancers more commonly exhibit *KRAS* mutations, which directly drive constant activation of the Ras/Raf/MAPK pathway, leading to unchecked cellular proliferation. Colorectal cancers more commonly show *TP53* mutations, which impair the cell’s ability to trigger damage to the DNA, cell cycle arrest, and apoptosis [16]. Similar to *KRAS* mutations, *PKD1* mutations in PCKD may also indirectly lead to dysregulation of the RAS-MAPK pathway [17]. This suggests that further research on understanding the genetic origins of this disease and its method of spreading will assist the creation of a framework to fight appendiceal malignancies.

## Conclusions

This case highlights several diagnostic complexities, ranging from the lack of clinical suspicion to the rarity of the disease and a comorbid condition. Unfortunately, these challenges obscured the diagnostic process to the extent that there are no more curative options for this patient. This patient’s unique presentation serves as a reminder to clinicians not to overlook appendiceal malignancies in patients with symptoms of appendicitis. 

While appendiceal carcinoma is a rare diagnosis, our case emphasizes that rarity does not equate to irrelevance. Each missed or delayed diagnosis not only represents a lost opportunity for expanded treatment options but also a missed chance to collect clinical data and awareness. Uncommon presentations can carry profound implications, and vigilance in the face of diagnostic uncertainty may be the only safeguard against overlooking rare but life-altering disease.

## References

[1] Yu M, Chuang W, Yu C (2016). Risk of cancer in patients with polycystic kidney disease: a propensity-score matched analysis of a nationwide, population-based cohort study. Lancet Oncol.

[2] Ruoff C, Hanna L, Zhi W, Shahzad G, Gotlieb V, Saif MW (2011). Cancers of the appendix: review of the literatures. ISRN Oncol.

[3] Dsouza R, Osueni A, Menon G (2025). Appendiceal tumors. StatPearls [Internet].

[4] Holowatyj N., Babyak R., Francis R. (2025). The presenting symptoms of patients with appendiceal malignant tumors (Abstract 824). J Clin Oncol.

[5] Skendelas JP, Alemany VS, Au V, Rao D, McNelis J, Kim PK (2021). Appendiceal adenocarcinoma found by surgery for acute appendicitis is associated with older age. BMC Surg.

[6] Cachat F, Renella R (2016). Risk of cancer in patients with polycystic kidney disease. Lancet Oncol.

[7] Sawaftah Z, Awashra A, Odah AB (2024). Adenocarcinoma of the appendix presenting as chronic small bowel obstruction: a case report. Radiol Case Rep.

[8] Misdraji J, Yantiss RK, Graeme-Cook FM, Balis UJ, Young RH (2003). Appendiceal mucinous neoplasms: a clinicopathologic analysis of 107 cases. Am J Surg Pathol.

[9] Mällinen J, Rautio T, Grönroos J (2019). Risk of appendiceal neoplasm in periappendicular abscess in patients treated with interval appendectomy vs follow-up with magnetic resonance imaging: 1-year outcomes of the peri-appendicitis acuta randomized clinical trial. JAMA Surg.

[10] Davidson GH, Flum DR, Monsell SE (2021). Antibiotics versus appendectomy for acute appendicitis - longer-term outcomes. N Engl J Med.

[11] McGory ML, Maggard MA, Kang H, O'Connell JB, Ko CY (2005). Malignancies of the appendix: beyond case series reports. Dis Colon Rectum.

[12] Racek AR, Rabe KG, Wick MJ, Psychogios A, Lindor NM (2011). Primary appendiceal mucinous adenocarcinoma in two first-degree relatives: case report and review. Hered Cancer Clin Pract.

[13] Tsai LW, Shih CM, Li SY, Tseng SH, Dubey R, Wu MS (2023). Susceptibility of developing renal and lung cancer in polycystic kidney disease: an evidence in reaching consensus. Euro J Can Care.

[14] Nozaki K, Ubara Y, Marui Y, Tomikawa S (2012). Mucinous cystadenoma of the appendix mimicking polycystic kidney disease. Int Braz J Urol.

[15] van den Boom AL, de Wijkerslooth EM, Mauff KA, Dawson I, van Rossem CC, Toorenvliet BR, Wijnhoven BP (2018). Interobserver variability in the classification of appendicitis during laparoscopy. Br J Surg.

[16] Holowatyj AN, Overman MJ, Votanopoulos KI (2025). Defining a 'cells to society' research framework for appendiceal tumours. Nat Rev Cancer.

[17] Distefano G, Boca M, Rowe I (2009). Polycystin-1 regulates extracellular signal-regulated kinase-dependent phosphorylation of tuberin to control cell size through mTOR and its downstream effectors S6K and 4EBP1. Mol Cell Biol.

